# Recent advances in craniopharyngioma pathophysiology and emerging therapeutic approaches

**DOI:** 10.3389/fendo.2025.1562942

**Published:** 2025-05-13

**Authors:** Clariano Pires de Oliveira Neto, Gilvan Cortês Nascimento, Sabrina da Silva Pereira Damianse, Manuel dos Santos Faria

**Affiliations:** ^1^ Post-Graduate Program in Health Sciences (PPGCS), Federal University of Maranhão (UFMA), São Luis, Brazil; ^2^ Service of Endocrinology, University Hospital of the Federal University of Maranhao (HUUFMA), São Luis, Brazil; ^3^ Research Group in Clinical and Molecular Endocrinology and Metabology (ENDOCLIM), Federal University of Maranhão (UFMA), São Luis, Brazil; ^4^ Clinical Research Center (CEPEC), University Hospital of the Federal University of Maranhao (HUUFMA), São Luís, Brazil; ^5^ Department of Medicine I, Federal University of Maranhao (UFMA), São Luis, Brazil

**Keywords:** craniopharyngioma, adamantinomatous craniopharyngioma, papillary craniopharyngioma, target therapies, precision medicine

## Abstract

Craniopharyngiomas are rare intracranial tumors originating from the Rathke’s pouch, affecting the sellar and parasellar regions. Despite their benign nature, they cause significant morbidity and mortality due to their proximity to vital structures such as the optic pathways and the hypothalamic-pituitary axis, resulting in endocrine, visual, neurological impairment, and hypothalamic syndrome. Classified into adamantinomatous (ACP) and papillary (PCP), these tumors differ in epidemiology, histology, and pathophysiology. ACP, the most common type, presents a bimodal peak incidence between 5 and 15 years of age and 45 and 60 years of age, while PCP is more restricted to adults. Traditional treatments such as surgery and radiotherapy face significant challenges, including high recurrence rates. Intracystic chemotherapy is used in monocystic ACP but with limited efficacy and adverse effects related to toxicity. Recent advances in molecular biology have introduced targeted therapies, such as BRAF and MEK inhibitors, which show potential benefits in craniopharyngioma patients, particularly in the PCP. For ACP, however, therapeutic outcomes remain limited despite advances in molecular understanding, including mutations in the *CTNNB1* gene and growth factors. Increasing investigation into the inflammatory microenvironment and immune response of these tumors presents new therapeutic possibilities and promising alternatives for tumor control, such as the use of anti-IL-6R, anti-VEGF agents and immune checkpoints inhibitors. This review aims to synthesize advancements in the pathophysiology of craniopharyngiomas and explore emerging therapeutic implications, focusing on precision medicine approaches for the management of this challenging disease.

## 1 Introduction

Craniopharyngiomas are rare intracranial epithelial tumors that develop from remnants of Rathke’s pouch, predominantly located in the sellar and parasellar regions (1, 2). Two main histological types are recognized: adamantinomatous craniopharyngioma (ACP) and papillary craniopharyngioma (PCP) (1). These tumors account for 1–3% of all primary intracranial tumors in adults and 5–10% of intracranial tumors in children, with an annual incidence ranging from 0.13 to 2 per 100,000 individuals and no gender predilection (2).

ACP is more common in children and young adults, displaying a bimodal age distribution (5–15 years and 45–60 years) and is frequently associated with somatic mutations in the *CTNNB1* gene encoding β-catenin. In contrast, PCP predominantly occurs in adults, especially in the fifth and sixth decades of life, and is associated with *BRAF V600E* mutations (1, 3).

Although histologically classified as WHO low grade I benign tumors, craniopharyngiomas exhibit complex clinical behavior and significant morbidity and mortality (4). Treatment typically involves a multidisciplinary approach combining surgery and radiotherapy to control the tumor while preserving hypothalamic and pituitary functions. Total resection is preferred when feasible, but subtotal resection followed by radiotherapy is often used due to proximity to critical neurovascular structures, especially in cases of hypothalamic invasion (3, 5). While total resection can provide local control, it is associated with a high risk of morbidity, including hypothalamic and pituitary dysfunction (6). Subtotal resection with radiotherapy offers comparable control rates with fewer complications (7, 8). Various radiotherapy modalities are effective, but may cause side effects like visual deterioration and endocrinopathies, depending on tumor characteristics and patient factors (9, 10). The limited efficacy and considerable side effects of traditional therapies underscore the need for innovative strategies to improve therapeutic outcomes and mitigate adverse effects (11).

In light of the limitations of conventional therapeutic approaches, the use of advanced omics technologies – such as next-generation sequencing (NGS) and single-cell transcriptomics (scRNA-seq) – has emerged as a promising strategy to unravel the molecular complexity of craniopharyngiomas. These approaches provide novel insights for developing more effective and personalized therapies. By enabling the identification of specific molecular targets and stratification of patients based on unique molecular profiles, they hold the potential to optimize treatment strategies and improve clinical outcomes (12, 13).

Recent advances in molecular biology have provided new insights into the genetic basis of craniopharyngiomas. Notably, the discovery of the *BRAF V600E* mutation in PCP has enabled the development of targeted therapeutic interventions. The use of BRAF inhibitors, such as vemurafenib and dabrafenib, either alone or in combination with MEK inhibitors, has shown efficacy in specific cases. This represents a paradigm shift in treatment, as these therapies offer more precise and less invasive alternatives compared to traditional approaches (14, 15).

Targeted therapies offer multiple advantages in the management of craniopharyngiomas. Unlike conventional methods, which often harm surrounding healthy tissues, these therapies specifically inhibit molecular pathways driving tumor growth. Consequently, they promote significant reductions in tumor size and improved clinical outcomes while minimizing complications and preserving essential neurological and endocrine functions. This highlights their potential for achieving better long-term results (14). Understanding the genetic alterations underlying craniopharyngiomas enables a personalized approach, tailoring treatment to the molecular profile of each patient. Furthermore, positive responses in cases refractory to conventional therapies reinforce the potential of these interventions as rescue options, offering new hope to patients with limited therapeutic alternatives (14, 16, 17).

This review aims to provide a comprehensive analysis of the current understanding of craniopharyngioma pathophysiology, with a particular emphasis on targeted therapies. By compiling and synthesizing available evidence, it seeks to highlight targeted therapies evaluated in craniopharyngioma patients with a pathophysiological basis, identify knowledge gaps, address emerging challenges, and delineate areas requiring further research to guide future directions.

## 2 Papillary craniopharyngioma

PCPs account for approximately 10% of craniopharyngiomas and almost exclusively occur in adults, predominantly in the fifth and sixth decades of life, with a mean age of 44.7 years (18, 19). These tumors typically present as large masses in the suprasellar region, often located above a preserved infundibulum or in the infundibulo-tuberal region of the third ventricle`s floor. Clinically, patients may present with visual deficits, hormonal alterations, memory impairment, and symptoms related to intracranial hypertension (19).

**Figure 1:**
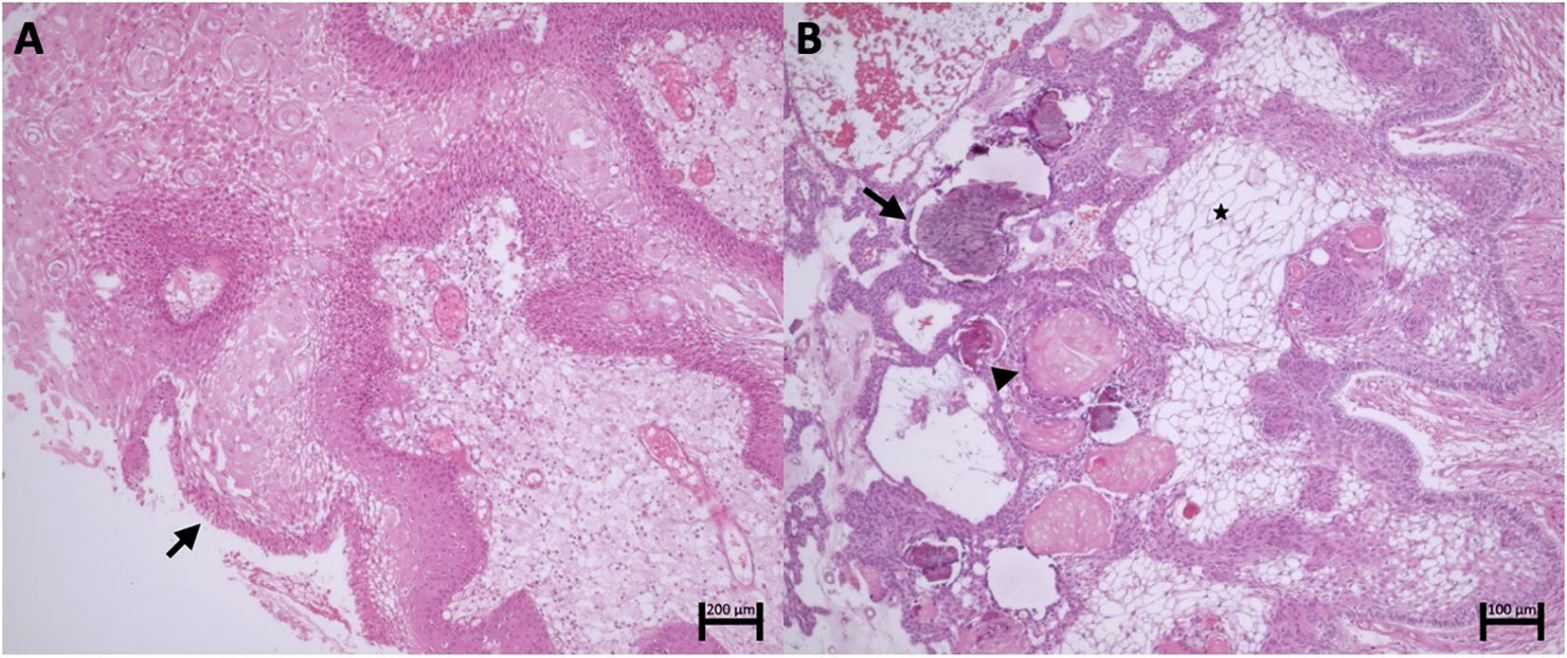
**(A)** Papillary craniopharyngioma. Fibrovascular connective axes forming papillae lined by well-differentiated squamous epithelium with palisading (arrow). **(B)** Adamantinomatous Craniopharyngioma. Islands and cords of epithelial cells with palisading delineating the stroma composed of stellate reticulum cells (asterisk). Clusters of anucleated squamous cells (arrowhead) and foci of calcification (arrow).

Macroscopically, PCPs consist of solid or mixed round masses, containing yellowish viscous cysts and rare calcifications. Histologically, these tumors are composed of well-defined neoplastic epithelium with cauliflower-shaped papillary projections, without infiltration into adjacent brain tissue. The histological component of PCPs includes pseudopapillae of mature squamous epithelium and an anastomosing fibrovascular stroma with fine capillaries and scattered immune cells, such as macrophages and neutrophils (18, 20) (**Figure 1**).

Unlike ACPs, PCPs do not present palisading reticular cells, wet keratin, or collagenous whorls, which are rare and small when present (17). Distinguishing PCPs from other suprasellar masses, such as Rathke’s pouch cysts, can be challenging (1).

**Figure 2:**
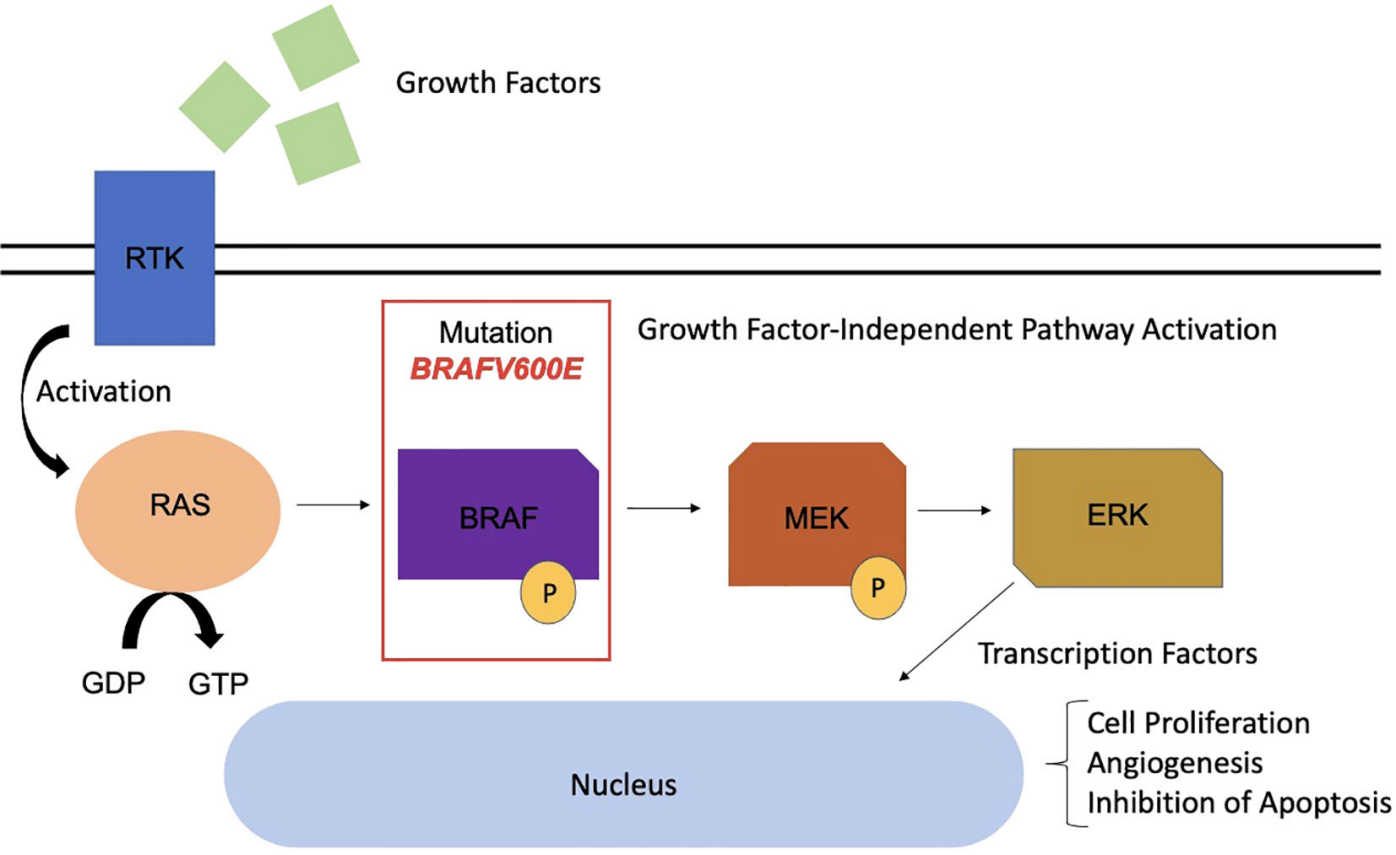
MAPK pathway with presence of *BRAF* mutation in the pathophysiology of papillary craniopharyngioma. RTK, Receptor tyrosine kinase; *RAS*, Rat Sarcoma; GDP, Guanosine Diphosphate; GTP, Guanosine Triphosphate; *BRAF*, B-Rapidly Accelerated Fibrosarcoma; MEK, Mitogen-Activated Protein Kinase; ERK, Extracellular Signal-Regulated Kinase.

In recent years, the genetic characterization of PCPs has advanced significantly. In 2014, the *BRAF V600E* mutation was identified through exome sequencing in three PCP samples, later confirmed in 36 of 39 additional samples (21, 22). The *BRAF V600E* mutation constitutively activates the MAPK/ERK pathway, an oncogenic signaling pathway that promotes the proliferation of SOX2+ embryonic cells, transforming them into tumor-initiating cells and stimulating processes such as angiogenesis and apoptosis inhibition (23, 24) (**Figure 2**).

**Figure 3:**
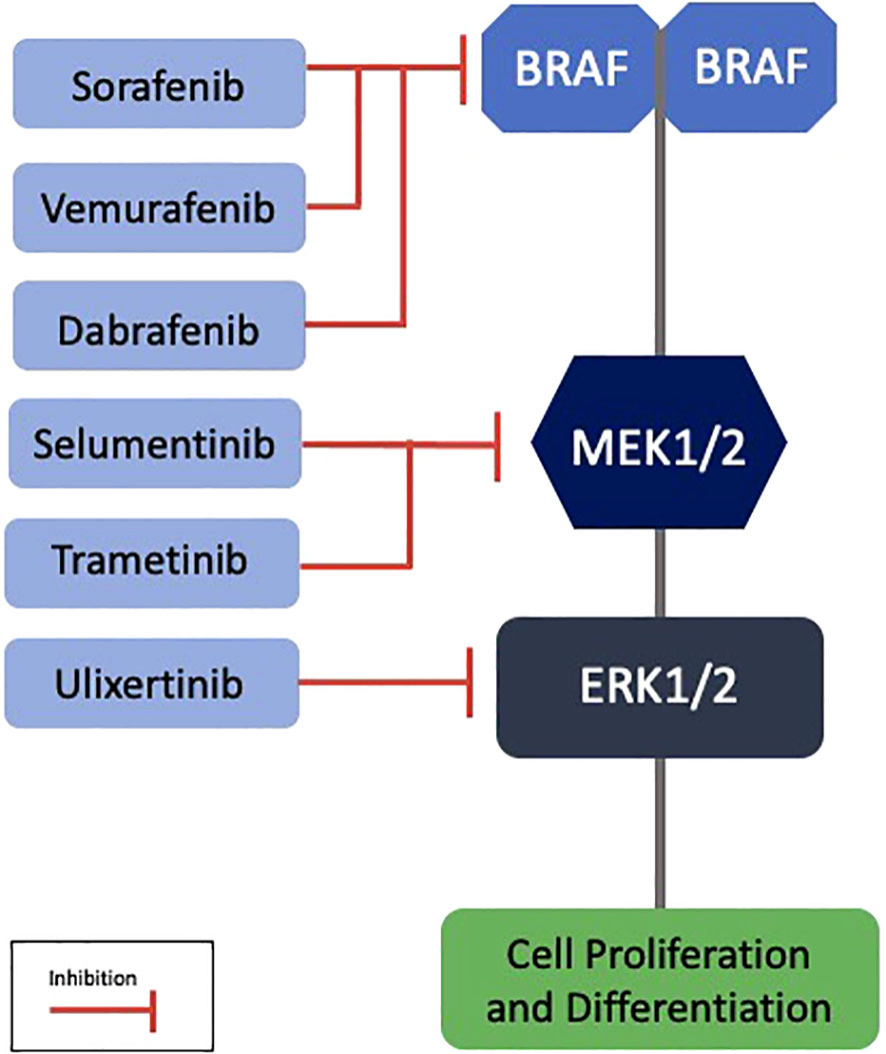
Targeted therapies in the MAPK pathway with potential application in papillary craniopharyngiomas. *BRAF*, B-Rapidly Accelerated Fibrosarcoma; MEK, Mitogen-Activated Protein Kinase; ERK, Extracellular Signal-Regulated Kinase.

The *BRAF V600E* mutation is identified in 81% to 100% of PCPs, serving as a genetic marker for this type (22, 24). Immunoreactivity for the VE1 antibody using immunohistochemistry confirms the presence of this mutation, while β-catenin remains localized to the cell membranes (21). This mutation is also observed in melanomas, and BRAF and MEK inhibitors such as vemurafenib, dabrafenib, and trametinib have revolutionized the treatment of these neoplasms, with promising results also for PCPs (25) (**Figure 3**).

Recent transcriptomic studies, including scRNA-seq have revealed distinct gene expression profiles in PCPs, marked by overexpression of genes associated with the MAPK pathway and Rathke’s pouch development (22, 26). Furthermore, characterization of the tumor microenvironment has revealed an immunosuppressive landscape enriched in regulatory T cells (13). These insights support the use of targeted therapy with BRAF and MEK inhibitors, which have yielded superior response rates in recurrent or unresectable PCPs compared to conventional treatment modalities (27, 28).

Transcriptomic data also reinforce the notion that PCPs are a molecularly homogeneous entity, with lower intratumoral heterogeneity than ACPs (13, 21, 22). Moreover, signals originating from fibroblasts and activating immune responses in neutrophils have been reported, highlighting the critical role of the tumor immune microenvironment in disease pathophysiology and the development of targeted therapies (26).

Recent studies report significant responses to treatment with BRAF and MEK inhibitors in cases of PCPs with the *BRAF V600E* mutation. In one of the first reported cases, there was an 85% reduction in the solid portions and 81% reduction in the cystic portions of the tumor following treatment with MEK/BRAF inhibitors (29). In a series with six patients, treatment with dabrafenib, trametinib, or vemurafenib, either alone or in combination, resulted in a reduction of 80% to 91% of the tumor masses, allowing for subsequent surgical or radiotherapeutic interventions (25).

The efficacy of these therapies with BRAF and MEK inhibitors has been extensively studied. In a phase II study, 94% of patients treated with the combination of vemurafenib and cobimetinib achieved a durable partial response, with a median tumor volume reduction of 91%. Disease progression-free survival was 87% at 12 months and 58% at 24 months, indicating significant tumor control (30). Dabrafenib, in combination with trametinib, has shown promise as a treatment option for papillary craniopharyngiomas with the BRAFV600E mutation. Studies and case reports suggest that this combination may lead to significant reductions in tumor volume and improvement in clinical symptoms, such as visual deficits and neurological dysfunctions (27, 29, 31). In a cohort study, the combination of dabrafenib and trametinib resulted in a partial response or better in 94% of patients, with an average tumor volume reduction of up to 91.8% in some treatment groups (27). A systematic review highlighted that treatment response may range from 24% to 100% volumetric reduction, with near-complete response observed in many cases (28) (**Table 1**).

**Table 1:** Targeted therapies for papillary craniopharyngioma (PCP).

Agent	n	Duration	Mechanism	Efficacy	Adverse effects
Vemurafenib + Cobimetinib Brastianos et al., 2023 (30)	16	8 cycles (28 days each)	BRAF and MEK Inhibition	Partial response in 94%, average tumor volume reduction of 91%, progression-free survival of 87% at 12 months and 58% at 24 months.	Rash, elevated creatine kinase, hyperglycemia
Dabrafenib + Trametinib Alcubierre et al., 2024 (27)	16	7.6 ± 5.3 months	BRAF and MEK Inhibition	Tumor volume reduction of 73.3% to 91.8%. Improvement in neurological symptoms.	Low-grade fever, fatigue, cough, peripheral edema, skin rash, anemia, elevated liver enzymes, verrucous keratoses, hyperglycemia

These findings strengthen the potential of targeted therapies in the management of PCPs, especially in patients refractory to traditional treatments. The accurate identification of the genetic mutations involved not only facilitates differential diagnosis but also opens new perspectives for personalized and more effective therapeutic approaches (21).

## 3 Adamantinomatous craniopharyngioma

ACPs represent 90% of craniopharyngiomas and can affect individuals of any age, showing a bimodal distribution with peaks between 5–15 and 45–60 years (32). The mean age at diagnosis in children under 15 years is 8.8 years (1, 18). Studies have shown no significant differences between pediatric and adult ACPs (24, 33).

These tumors are commonly cystic, with calcifications and cholesterol-rich contents resembling “motor oil” (34). Their irregular margins, with palisading basal layers infiltrating into surrounding tissues in finger-like projections, are surrounded by intense gliosis, complicating surgical identification of planes (35). The heterogeneous tumor epithelium is juxtaposed with stellate reticulum and wet keratin nodules, composed of ghost and squamous cells, often associated with calcifications, cholesterol crystals, and hemosiderin deposits from chronic hemorrhage (1) (**Figure 1**).

In their pathophysiology, the Sonic Hedgehog (SHH) pathway is highly active, particularly in cell clusters and palisaded basal layers marked by Ki67 positivity (36, 37). The SHH pathway, linked to pituitary embryogenesis, appears to sustain tumor stem cells and promote tumor growth, infiltration, and angiogenesis via autocrine and paracrine mechanisms (38–40).

The pathogenesis of ACPs involves somatic mutations in the *CTNNB1* gene, which encodes β-catenin (41). Normally, the Wnt pathway regulates crucial processes like growth and metabolism, with β-catenin localized to cell membranes (42). β-catenin also participates in adhesion complexes with E-cadherin, maintaining cellular architecture (43). Activating mutations in *CTNNB1* are reported with prevalence rates ranging from 16% to 100%, depending on sequencing techniques (1, 17, 44). The heterogeneity in the prevalence of *CTNNB1* mutations may also reflect the complex cellular composition of the tumor, which includes distinct neoplastic and non-neoplastic subpopulations such as senescent cells, tumor germ cells, and cells with different cytokeratin (CK) expression profiles (12).

**Figure 4:**
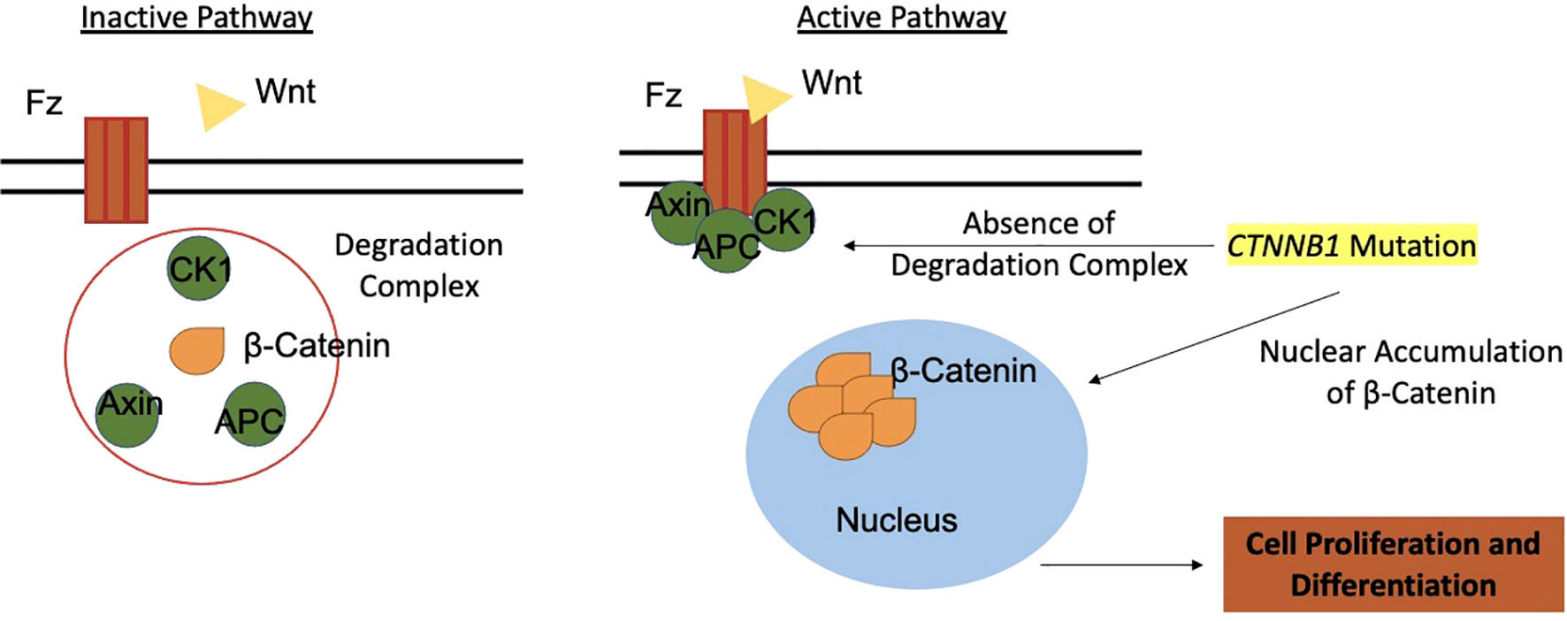
The Wnt pathway and its role in tumorigenesis of adamantinomatous craniopharyngiomas. Wnt, Wingless-Integration site; Fz, Frizzled receptor; CK1, Casein Kinase 1; APC, Antigen-presenting Cell; AXIN, Axin protein.

Mutations in *CTNNB1* affect exon 3, encoding β-catenin’s degradation complex, leading to aberrant nucleocytoplasmic accumulation in 96% of ACPs (39). This accumulation hyperactivates the Wnt pathway, critical for cell proliferation and pituitary embryogenesis, as evidenced by targets like *AXIN2*, *LEF1*, and *BMP4* (37, 45). In ACPs, β-catenin accumulates in small whorled clusters or near infiltrative edges, acting as signaling centers for cell proliferation and differentiation (45, 46) (**Figure 4**).

*CTNNB1* mutations are specific to ACPs and absent in other sellar region tumors, such as PCPs (17, 46, 47). However, simultaneous mutations in *CTNNB1* and *BRAF* have been identified in tumors with mixed adamantinomatous and papillary features (48). Wnt pathway hyperactivation correlates with aggressive disease and poorer overall survival rates (49).

Further studies identified fibroblast growth factors (FGFs), bone morphogenetic proteins (BMPs), and transforming growth factor-β (TGF-β) as mediators of tumor growth in ACPs (39, 45). Activation of the epidermal growth factor receptor (EGFR) stabilizes β-catenin in tumor cells coexpressing fascin, an actin-binding protein linked to adhesion, migration, invasion, and cytoskeletal reorganization. Fascin expression correlates with invasive growth and poor prognosis in ACP patients (50).

Moreover, reports suggest MAPK/ERK pathway activation in ACPs, with FGF, EGF and ERK1/2 expression colocalized with Ki67 in palisaded epithelium. Ex vivo experiments on ACPs treated with the MEK inhibitor trametinib demonstrated increased apoptosis and reduced cell proliferation, highlighting the relevance of this pathway (51).

Markers like CK, particularly CK8 and CK18, are elevated in β-catenin-positive cells, indicating dedifferentiation and tumor progression. Claudin-1 (CLDN1), a tight junction component, is downregulated in β-catenin-positive clusters and finger-like projections, suggesting a role in invasiveness. Claudins also influence cyst formation through fluid accumulation from endothelial leakage, impairing cellular adhesion (52).

Inflammation plays a central role in ACP pathophysiology. Cholesterol crystals from cystic components can stimulate IL-1β secretion, triggering immune activation (53). Spatial transcriptomic analyses have demonstrated that this inflammatory response involves heterogeneous immune cell infiltration, including tumor-associated macrophages (TAMs) with both M1 and M2 polarization. These processes may be modulated by interactions between tumor cells and the lipid-rich microenvironment (13).

Treating ACPs remains challenging, with no clear consensus on the optimal therapeutic approach. Since β-catenin is critical for normal processes, nonspecific inhibitors could harm healthy tissues (54). Therapeutic approaches vary widely, reflecting the complexity of managing ACPs. Interferon-α (INF-α) shows mixed outcomes, ranging from complete response to disease progression (55, 56). Pegylated Interferon-α-2b (INF-peg-α-2b) appears more effective, with studies showing complete responses or significant cystic reduction (57, 58). Other inflammatory mediators like tocilizumab (anti-IL-6R) and bevacizumab (anti-VEGF) are promising, with reports of significant tumor volume reduction after recurrences (59–61). Understanding the role of the MEK/ERK pathway in ACPs may also enable therapeutic targeting. MEK inhibitor binimetinib reduced tumor volume by over 95% after 21 months of treatment (62) (**Table 2**).

**Table 2:** Targeted therapies for adamantinomatous craniopharyngiomas (ACP).

Agent	n	Duration	Mechanism	Efficacy	Adverse effects
Interferon-α Kilday et al., 2017 (97)	56	5,1 years	Immunomodulation	Varied response (ranging from complete response to disease progression)	Fever, chills, myalgias, hypotension, lethargy, nausea, vomiting, elevated transaminases, thrombocytopenia, seizures, hyperpigmentation, weight loss
Pegylated Interferon-α-2b Goldman et al., 2020 (58)	19	108 weeks	Immunomodulation	Partial response in 28%. Median disease-free survival of 19.5 months.	Nausea, fever, constitutional symptoms, elevated transaminases, thrombocytopenia, fatigue, neutropenia
Tocilizumab Grob et al., 2019 (60)	2	6 months	Anti-IL-6R	Reduction of cystic volume and disease stability	Neutropenia
Bevacizumab De Rosa et al., 2023 (61)	1	6 weeks	Anti-VEGF	Partial response (66% tumor volume reduction after 3 months)	Not reported
Binimetinib Patel et al., 2021 (62)	1	8 months	MEK Inhibition	Disease stability	Skin rash, nail dystrophy, hyponatremia, venous stasis, fatigue, daytime sleepiness, weight gain.

Despite these advances, therapeutic responses remain highly variable. The molecular and cellular heterogeneity of ACPs – revealed through single-cell and spatial transcriptomic approaches – emphasizes the need for individualized treatments tailored to each tumor’s unique profile (63). Integrated multi-omics analyses encompassing genomic, transcriptomic, proteomic, and spatial data, combined with detailed microenvironment characterization, represent a promising strategy for identifying predictive biomarkers, stratifying patients, and developing more precise and effective therapies (12). A deeper understanding of the cellular and molecular crosstalk within the tumor microenvironment—including the roles of senescent cells, tumor germ cells, and macrophage polarization—may ultimately pave the way for innovative therapeutic approaches in ACP treatment (13, 64–67).

## 4 Inflammatory mediators and immune response

Inflammation plays a significant role in the pathophysiology of craniopharyngiomas, influencing both the biological behavior of the tumor and the clinical prognosis of patients (11, 51). Several inflammatory mediators, including interleukins (IL-6, IL-8, IL-10), tumor necrosis factor (TNF), and chemokines (CXCL12, CXCR4), are implicated in tumor progression and the pathological inflammatory microenvironment of these tumors (68). The presence of a pronounced inflammatory response correlates with a higher incidence of hypopituitarism and a lower likelihood of recurrence-free survival (68). Moreover, β-catenin, frequently mutated in adamantinomatous craniopharyngiomas (ACPs), not only participates in Wnt signaling but also interacts with inflammatory mediators, amplifying cytokine and chemokine production in the tumor microenvironment. This process establishes a vicious cycle wherein inflammation perpetuates cellular proliferation and fosters tumor recurrence (69).

### 4.1 Interleukins

Interleukins, such as IL-6 and IL-8, are often found in elevated levels within tumor tissues and are associated with the promotion of a pro-tumorigenic microenvironment (70). IL-6 is known for its ability to enhance tumor invasion and angiogenesis while mediating communication between tumor cells and immune cells, such as tumor-associated macrophages, which can increase tumor aggressiveness. This occurs via epithelial-mesenchymal transition (EMT), a process that boosts the migratory capacity of tumor cells, facilitating local invasion (71). Furthermore, elevated levels of IL-6 and its receptor (IL-6R) suggest that this signaling pathway could be a potential therapeutic target, as demonstrated in studies exploring the use of tocilizumab, a monoclonal antibody that blocks IL-6R, to reduce cystic volume in ACP patients (60).

IL-8 also plays a significant role in the tumor microenvironment of craniopharyngiomas, contributing to local invasion and adhesion to surrounding tissues (72). Studies indicate that IL-8, alongside other pro-inflammatory cytokines such as IL-6, is elevated in the plasma of patients and brain tumor tissues (68, 73). IL-8 is further recognized for its pro-tumorigenic functions across various cancers, including promoting angiogenesis, increasing cellular proliferation and survival, and facilitating cell migration (72). Although specific literature on craniopharyngiomas is limited, IL-8’s role in other tumors suggests that it may contribute to tumor progression through similar mechanisms by modulating the tumor microenvironment (68, 74).

Vascular Endothelial Growth Factor (VEGF), a key mediator of angiogenesis, is an essential component in craniopharyngioma pathophysiology, driving the formation of new blood vessels that supply the tumor with nutrients and oxygen (75). It is present in epithelial cells of both ACP and PCP, and microvascular density related to VEGF expression correlates with tumor recurrence, suggesting the prognostic value of angiogenesis extent. VEGF is also linked to cyst formation in craniopharyngiomas, with its expression appearing to correlate with tumor size (76). Regulated by β-catenin and other molecular pathways, VEGF interacts with matrix metalloproteinases (MMPs), such as MMP-9, to remodel the extracellular matrix and facilitate tumor invasion. This remodeling may be critical for craniopharyngioma progression. MMP-1, for instance, can enhance VEGF-2R expression on endothelial cells, promoting cellular proliferation and angiogenesis via intracellular signaling pathways (77). Evidence also suggests that MMPs may induce the production of the anti-apoptotic protein Bcl-2, which regulates tumor growth and recurrence through autocrine and paracrine mechanisms (78). Dysregulation of apoptosis-related genes, including members of the Bcl-2 family, seems to play a significant role in the pathogenesis of pituitary adenomas (79). Although the specific role of MMPs in craniopharyngiomas remains underexplored, their involvement in angiogenesis and VEGF modulation suggests they contribute to tumor pathogenesis.

The interplay between IL-6 and VEGF establishes a pathological axis that amplifies inflammation and vascularization in the tumor microenvironment. IL-6 can upregulate VEGF expression, creating a feedback loop that intensifies these processes (80). This interaction also contributes to resistance to conventional therapies, highlighting the need for integrated approaches to disrupt this cycle.

While studies on anti-IL-6 and anti-VEGF therapies in craniopharyngiomas remain limited, preliminary evidence suggests therapeutic potential. A study reported the combined use of tocilizumab (anti-IL-6R) and bevacizumab (anti-VEGF) in two patients with recurrent cystic ACPs, resulting in significant responses, including reduced cystic tumor burden and tumor stability, suggesting a viable therapeutic alternative (60).

Inflammatory mediators also play a significant role in PCPs, influencing both tumor invasiveness and the immune microenvironment. The use of preoperative inflammatory markers, such as neutrophil counts and the neutrophil-to-lymphocyte ratio, can aid in the differential diagnosis of PCPs from other sellar region tumors, with higher levels observed in PCPs. These tumors exhibit high immune infiltration but with low activity, attributed to extensive neutrophil infiltration that creates an inactive immune microenvironment (81). Furthermore, elevated IL-6 expression is positively correlated with PCP tumor invasion into the hypothalamus, suggesting that these inflammatory mediators could serve as potential therapeutic targets to prevent tumor invasion (82).

### 4.2 Chemokines

Chemokines from the CXC and CC families play significant roles in craniopharyngioma pathophysiology, particularly in ACP. The CXCL12/CXCR4 axis is specifically implicated in tumor progression. Overexpression of CXCL12 and CXCR4 in ACP promotes tumor cell proliferation, migration, and invasion, primarily via the activation of the PI3K/AKT signaling pathway (83, 84). This pathway is critical in regulating cell growth and survival, indicating that the CXCL12/CXCR4 axis contributes to tumor aggressiveness. These chemokines are also recognized for their capacity to influence the tumor microenvironment by modulating angiogenesis and immune responses, potentially affecting tumor progression (85). Although the impact of other CC and CXC chemokines on craniopharyngiomas is less studied, their roles in other cancers suggest similar contributions to tumor growth and invasion (86).

### 4.3 Immune checkpoint

Immune checkpoints are essential molecules and pathways in the immune system that regulate immune responses, preventing them from becoming destructive to the body’s healthy cells. These include inhibitory receptors and ligands that play a crucial role in modulating T-cell activation and function. Under normal conditions, these checkpoints help maintain immune tolerance and prevent collateral damage during antimicrobial immune responses (87). In the context of cancer, tumor cells can exploit these checkpoints to evade immune destruction. This occurs when proteins on the surface of T-cells, known as immune checkpoint proteins, recognize and bind to partner proteins on cancer cells, sending inhibitory signals that suppress the immune response (88). Therapies that block these checkpoints, known as immune checkpoint inhibitors, have shown significant efficacy in restoring T-cell capability to attack tumor cells, leading to robust tumor regressions (89).

Immune checkpoint inhibitors have demonstrated significant potential in the treatment of various cancer types. Blocking programmed cell death protein 1 (PD-1) and its ligand (PD-L1) with agents such as nivolumab and pembrolizumab has improved survival in cases of melanoma and non-small cell lung adenocarcinoma (90, 91). The availability of these agents, combined with their relatively favorable side effect profiles, has prompted numerous studies investigating their efficacy across various tumor types.

Recent studies have demonstrated PD-L1 expression in the epithelial cells lining cysts and intrinsic PD-1 expression in cell clusters in ACPs with β-catenin overexpression. These clusters play a central role in tumor growth in ACPs through various mechanisms, making PD-1 targeting a promising therapy (92, 93). Two more studies also demonstrated elevated PD-L1 expression in ACPs, revealing this as a potential therapeutic target in craniopharyngiomas (94, 95).

## 5 Ongoing clinical trials

**Figure 5:**
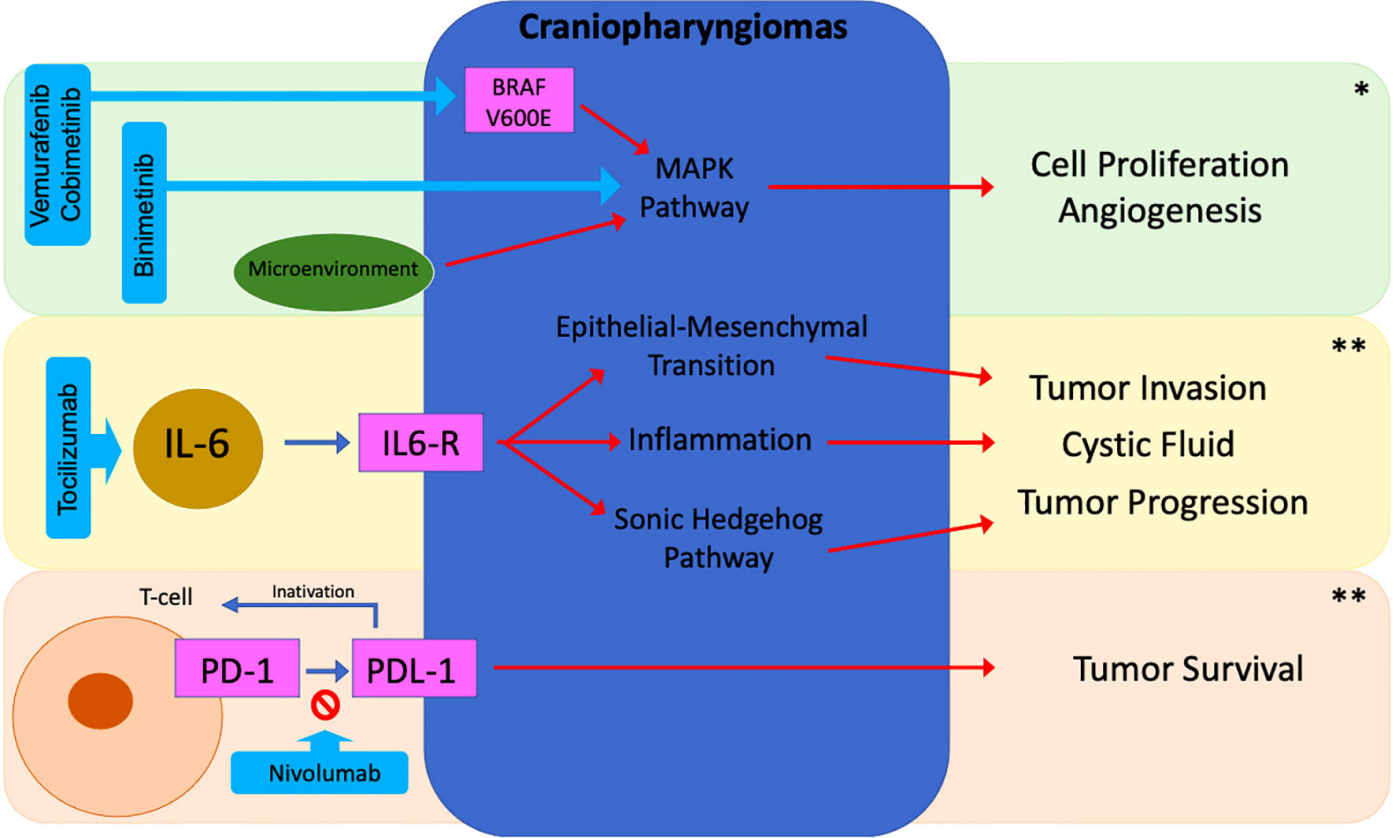
Potential targeted therapies in craniopharyngiomas. *ACP/PCP; **ACP; *BRAF*, B-Rapidly Accelerated Fibrosarcoma; IL-6, Interleukin 6; PD-1, Programmed Cell Death Protein 1; PD-L1, Programmed Cell Death Protein Ligand 1.

In recent years, the multi-omic characterization of craniopharyngiomas has provided significant insights into their pathogenesis, driving clinical trials aimed at developing more personalized and lower-risk therapeutic approaches. Ongoing studies with binimetinib (NCT05286788), tocilizumab (NCT05233397), and nivolumab (NCT05465174) for ACP as well as vemurafenib and cobimetinib (NCT03224767) for the PCP, offer promising therapeutic alternatives and may help define optimal patient profiles (96) (**Figure 5**) (**Table 3**). However, some challenges still remain regarding the optimal timing for introducing these treatments—whether as neoadjuvant therapy to reduce tumor volume before surgery or as adjuvant therapy to minimize recurrence and improve disease control—as well as the appropriate duration of treatment. Furthermore, uncertainties persist regarding their use as monotherapy, in combination with other agents, or in conjunction with surgery and radiotherapy, underscoring the need for further studies to refine the management of these rare tumors.

**Table 3:** Targeted therapies for inflammatory mediators and for immune response.

Inflammatory and Immune Target	Mechanism	Therapeutic Potential
IL-6 (60)	Tumor invasion, angiogenesis, immune cell/tumor communication, and epithelial-to-mesenchymal transition (EMT).	Anti-IL-6R monoclonal antibodies (e.g., tocilizumab) reduce tumor invasion and cystic volume.
IL-8 (68)	Angiogenesis, cell proliferation, migration, and cell adhesion.	Blocking IL-8-related pathways to reduce local invasion and angiogenesis.
VEGF (61)	Angiogenesis and cyst formation; interacts with MMPs for extracellular matrix remodeling.	VEGF inhibitors (e.g., bevacizumab) to reduce angiogenesis and tumor growth.
CXCL12/CXCR4 (83, 84)	Tumor progression, migration, invasion, and angiogenesis through activation of CXCR4 signaling pathways.	Limit tumor progression and invasion.
PD-1/PD-L1 (87)	Inhibits the immune response, allowing tumor evasion.	Immune checkpoint inhibitors (e.g., nivolumab, pembrolizumab) to reactivate the immune system against the tumor.

## 6 Conclusion

Recent advances in understanding the pathophysiology of craniopharyngiomas provide a promising foundation for developing more effective therapeutic strategies. Given the limitations of conventional approaches, targeted therapies, such as BRAF and MEK inhibitors for the PCP, have shown encouraging results. Recently, the growing understanding of the inflammatory behavior and immune response of these tumors has highlighted the therapeutic potential of anti-IL-6R and anti-VEGF agents and immune checkpoints inhibitors, signaling a promising future for the application of precision medicine in this field in both, APC and PCP.

Significant gaps still remain, particularly in managing more aggressive or resistant tumors. Robust clinical trials and interinstitutional collaborations are crucial to validate these therapies on a larger scale and standardize treatment protocols. Additionally, identifying predictive biomarkers and elucidating molecular interactions within the tumor microenvironment may offer new therapeutic opportunities, improve prognostic outcomes, and reduce the morbidity and mortality associated with craniopharyngiomas.
